# Sleep Disturbance and Quality of Life in Rheumatoid Arthritis: Prospective mHealth Study

**DOI:** 10.2196/32825

**Published:** 2022-04-22

**Authors:** John McBeth, William G Dixon, Susan Mary Moore, Bruce Hellman, Ben James, Simon D Kyle, Mark Lunt, Lis Cordingley, Belay Birlie Yimer, Katie L Druce

**Affiliations:** 1 Centre for Epidemiology Versus Arthritis University of Manchester Manchester United Kingdom; 2 National Institute for Health Research Manchester Musculoskeletal Biomedical Research Centre Central Manchester University Hospitals National Health Service Foundation Trust Manchester United Kingdom; 3 uMotif London United Kingdom; 4 Sleep and Circadian Neuroscience Institute Nuffield Department of Clinical Neurosciences University of Oxford Oxford United Kingdom; 5 Division of Musculoskeletal and Dermatological Sciences Manchester University Manchester United Kingdom

**Keywords:** mobile health, sleep, rheumatoid arthritis, pain, fatigue, mood, sleep disturbance, HRQoL, quality of life, health-related quality of life, QoL, sleep efficiency, WHOQoL-BREF, mobile phone

## Abstract

**Background:**

Sleep disturbances and poor health-related quality of life (HRQoL) are common in people with rheumatoid arthritis (RA). Sleep disturbances, such as less total sleep time, more waking periods after sleep onset, and higher levels of nonrestorative sleep, may be a driver of HRQoL. However, understanding whether these sleep disturbances reduce HRQoL has, to date, been challenging because of the need to collect complex time-varying data at high resolution. Such data collection is now made possible by the widespread availability and use of mobile health (mHealth) technologies.

**Objective:**

This mHealth study aimed to test whether sleep disturbance (both absolute values and variability) causes poor HRQoL.

**Methods:**

The quality of life, sleep, and RA study was a prospective mHealth study of adults with RA. Participants completed a baseline questionnaire, wore a triaxial accelerometer for 30 days to objectively assess sleep, and provided daily reports via a smartphone app that assessed sleep (Consensus Sleep Diary), pain, fatigue, mood, and other symptoms. Participants completed the World Health Organization Quality of Life-Brief (WHOQoL-BREF) questionnaire every 10 days. Multilevel modeling tested the relationship between sleep variables and the WHOQoL-BREF domains (physical, psychological, environmental, and social).

**Results:**

Of the 268 recruited participants, 254 were included in the analysis. Across all WHOQoL-BREF domains, participants’ scores were lower than the population average. Consensus Sleep Diary sleep parameters predicted the WHOQoL-BREF domain scores. For example, for each hour increase in the total time asleep physical domain scores increased by 1.11 points (*β*=1.11, 95% CI 0.07-2.15) and social domain scores increased by 1.65 points. These associations were not explained by sociodemographic and lifestyle factors, disease activity, medication use, anxiety levels, sleep quality, or clinical sleep disorders. However, these changes were attenuated and no longer significant when pain, fatigue, and mood were included in the model. Increased variability in total time asleep was associated with poorer physical and psychological domain scores, independent of all covariates. There was no association between actigraphy-measured sleep and WHOQoL-BREF.

**Conclusions:**

Optimizing total sleep time, increasing sleep efficiency, decreasing sleep onset latency, and reducing variability in total sleep time could improve HRQoL in people with RA.

## Introduction

### Background

People living with rheumatoid arthritis (RA), a long-term progressive autoimmune disease, experience a significantly reduced health-related quality of life (HRQoL), which can be characterized as the impact a condition has on physical, emotional, and social well-being. People with RA have poorer HRQoL compared with patients with other rheumatic diseases [1] and the general population [2]. There are likely numerous causes for poor HRQoL. RA disease activity is a major contributor to lower HRQoL, although HRQoL remains significantly lower than that of the general population, even in those with well-controlled disease. Sleep disturbances are common in RA [3] and have been identified by patients as a possible driver of low HRQoL [4-6].

**Figure 1:**
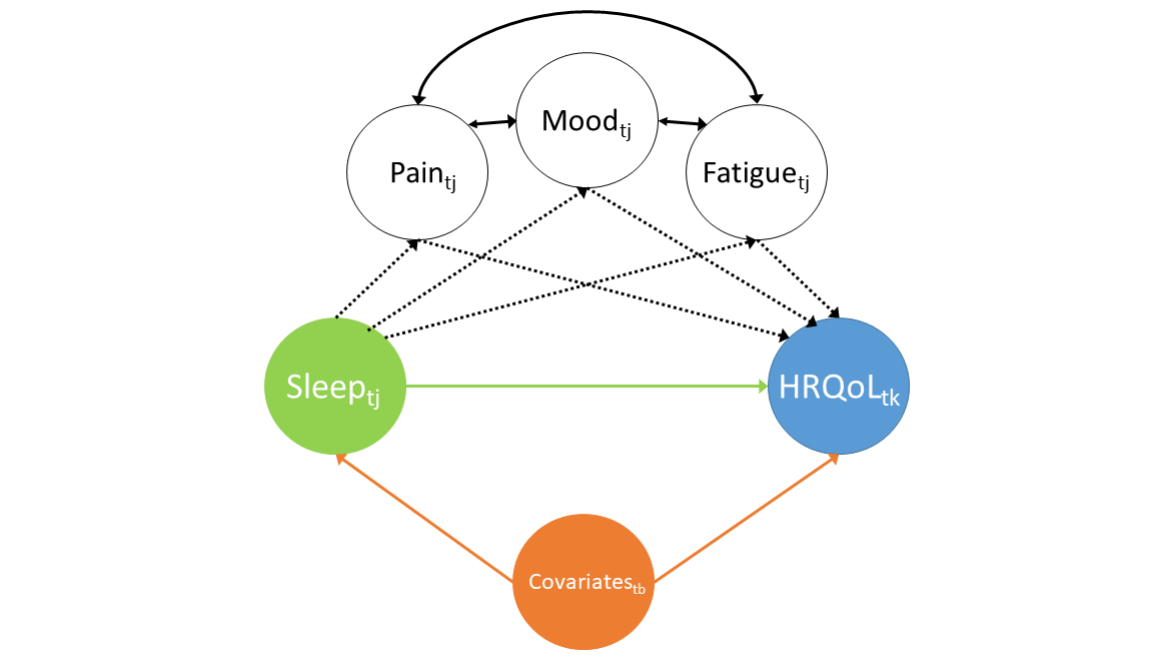
Directed acyclic graph for the relationship among sleep, health-related quality of life (HRQoL), pain, mood, and fatigue. The likelihood of reporting a particular level of health-related quality of life at days 10, 20, or 30 (HRQoL_tk_) is directly predicted by sleep (Sleep_tj_) in the previous 10 days (green arrow) as well as the effect of Sleep_tj_ acting through pain (Pain_tj_), mood (Mood_tj_), and fatigue (Fatigue_tj_) in the previous 10 days (black dashed lines). Pain, fatigue, and mood increase the likelihood of each other (black solid lines). The relationship may be confounded by covariates measured at baseline including age, sex, and disease severity (Covariates_tb_; orange arrows).

Studies of sleep in RA have reported less total sleep time, more waking periods after sleep onset, higher levels of nonrestorative sleep [5], and increased periods of mini arousal [4]. During a disease flare, people with RA experience more fragmented sleep, shorter total sleep time, and lower sleep efficiency [7-9]. However, few studies have determined the relationship between sleep variables and HRQoL and understanding whether these sleep disturbances reduce HRQoL remains a challenge. First, sleep is a multifaceted behavior comprising both objective and subjective components [10]. Thus, a comprehensive assessment of sleep health requires measurement of objective and self-reported sleep domains, including appraisals of sleep quality and quantitative estimates of sleep continuity and duration [11]. Despite this, it is only subjective sleep which has been commonly measured in epidemiological studies because, historically, it has been difficult to objectively measure sleep outside artificial laboratory settings. Many studies have also tended to be cross-sectional, despite the high degree of between-day variability, with individuals fluctuating between good and poor sleep states [12]. In addition, sleep disturbance increases the severity of common RA symptoms, including pain, mood, and fatigue, which are known to cause poor HRQoL [13]. Whether poor HRQoL in people with RA is a direct effect of sleep disturbance or an indirect consequence of changes in the severity of pain, mood, and fatigue (Figure 1) is not clear. Understanding these relationships would inform the development of interventions to improve HRQoL. Capturing these complex time-varying data with sufficiently high resolution to understand these relationships has been made possible by the widespread availability and use of mobile health (mHealth) technologies. mHealth technologies, including smartphone apps and wearables, allow frequent and repeated remote collection of patient-generated symptoms and other health data and objective assessments of sleep [14].

### Objectives

This prospective mHealth study tested the hypothesis that sleep disturbance (both absolute measures and variability) in people with RA would predict poor HRQoL. We then tested whether any observed relationship was explained by the effect of sleep on pain, mood, and fatigue severity.

## Methods

### Overview

The quality of life, sleep, and rheumatoid arthritis (QUASAR) study collected daily data from people living with RA for 30 days. The participants completed a baseline questionnaire and wore a triaxial accelerometer to assess sleep over the 30-day study period. On a patient co-designed smartphone app (Figure S1 in Multimedia Appendix 1) developed in collaboration with uMotif, participants completed a daily sleep diary; provided daily reports of the severity of their pain, fatigue, and mood; and completed a quality of life questionnaire every 10 days after baseline. QUASAR has been described in detail elsewhere [14], and the methods are summarized in the following sections.

### Participants

Eligible participants included those aged ≥18 years, with RA (classified as self-reported clinical diagnosis of RA and currently using disease-modifying antirheumatic drugs [15]), who had access to an Android or iPhone operating system (Apple Inc) smartphone or tablet, and who were not employed in shift work.

### Recruitment

Participants were recruited from May 1, 2017, to July 13, 2018, via an email sent to people registered on the electronic mailing list of the National Rheumatoid Arthritis Society, a UK-wide patient organization. The email contained an electronic study information pack, which included a participant information sheet, a copy of the study consent form, and a link to complete a web-based screening questionnaire. The screening questionnaire collected data on study eligibility criteria, contact information, and consent for further contact. Eligible participants were telephoned after at least 24 hours of questionnaire submission to discuss the project. Verbal consent was obtained, and a study pack (written consent form, baseline questionnaire, actigraph, and study instructions) was mailed to participants in time for the agreed study start date.

### Data Collected

#### Baseline Questionnaire

Data were collected on sex (male or female), date of birth (day, month, and year), and date of RA diagnosis (month and year); BMI (self-reported weight in kilograms/height in meters^2^) categorized as underweight (<18.5 kg/m^2^), healthy (18.5-24.9 kg/m^2^), overweight (24.9-30 kg/m^2^), or obese (>30 kg/m^2^); marital status (single, married or with partner, or separated); smoking (past, never, or current smoker); average weekly alcohol consumption (none: 0 units, moderate: 1-14 units, and high: ≥15 units); total number of medication types (range 0-4 from categories of painkillers, disease-modifying antirheumatic drugs, sleep medications, and others [free text]); and Index of Multiple Deprivation (English, 2015; Scottish, 2016; or Welsh, 2019, as appropriate) derived from the first part of participants’ postcodes.

Baseline sleep quality was assessed using the Pittsburgh Sleep Quality Index (PSQI) [16] (score range 0-21, higher scores indicating worse sleep quality). Insomnia was assessed using the Sleep Condition Indicator (SCI) [17], and participants reported physician-diagnosed obstructive sleep apnea (OSA) and restless leg syndrome (RLS). Anxiety was measured using the anxiety subscale of the Hospital Anxiety and Depression Scale (score range: 0-21; categorized as *not a case*: 0-8, *borderline case*: 8-11, and *case*: 11-21) [18].

Disease severity was assessed using the Routine Assessment of Patient Index Data 3 (RAPID-3) [19]. The 15-item RAPID-3 measures three domains: physical function, pain, and global health in the past week. The first 10 items of the physical function domain were scored, transformed into a 0.3-10 scale and summed with the pain and global health domains to produce an overall score of 0-30. RAPID-3 scores are correlated with the disease activity score 28 and clinical disease activity index in clinical trials and clinical care [20].

#### Sleep Assessments

##### Consensus Sleep Diary

Each morning at 8 AM, participants were prompted via an alert in the study app to complete the 10-item Consensus Sleep Diary (CSD), which assesses the quantity and quality of sleep. CSD is widely considered the gold standard sleep diary [21]. The CSD variables were *time taken to fall asleep* (minutes), *total time asleep* (hours), and *sleep efficiency* (proportion of in-bed time spent sleeping). The CSD also assessed *sleep quality* (5-point Likert scale, ranging from 1 [*very poor*] to 5 [*very good*]) and *feeling refreshed* on awakening (5-point Likert scale, ranging from 1 [*not at all rested*] to 5 [*very-well rested*]).

##### Actigraphy

Participants were asked to wear the MotionWatch 8 actigraphy monitor (CamNtech), a Conformitè Europëenne–marked Class 1 medical device, on their nondominant wrist 24 hours a day for 30 days. MotionWatch 8 was configured to capture limb or bodily movements in 30-second epochs using a triaxial accelerometer. Actigraphy has been shown to provide reliable estimates of sleep compared with polysomnography [22]. CamNtech proprietary software was used to extract the sleep parameters of interest. Running the software requires the time participants get in to bed and out of bed in each 24-hour period to be recorded. In-bed and out-of-bed times were determined either via self-reported times in the CSD or by manual screening of actigraphy data (if CSD data were missing). Where manual screening took place, in-bed times were defined as the time of peak of activity count data immediately before continuous activity ceased for the day, and out-of-bed time was defined as the time of trough of activity count data immediately before continuous activity began for the subsequent day. To assess reliability, 20.1% (54/268) of the data streams were inspected by 2 raters. The actigraph sleep variables were *time taken to fall asleep* (minutes), *total time asleep* (hours), *sleep efficiency* (proportion of in-bed time spent sleeping), and *fragmentation index* (the number of interruptions of sleep by physical movement with higher scores indicating more fragmented sleep).

### Pain, Fatigue, and Mood

Participants were prompted once in the morning at 8 AM and once in the evening at 6 PM to complete the uMotif interface within the study app (Figure S1 in Multimedia Appendix 1) to report the presence and severity of their pain, mood, and fatigue on a 5-point ordinal scale. Pain and fatigue severity were scored from 1 (*none*) to 5 (*very severe*), and mood was scored from 1 (*depressed*) to 5 (*very happy*).

### HRQoL Measurements

Participants completed the World Health Organization Quality of Life-Brief (WHOQoL-BREF) scale [23] using the study app at baseline and on days 10, 20, and 30. The recall period was 10 days to capture the changes since the previous assessment. Noncompleters received a reminder text to complete the assessment within 5 days of the original completion date. The WHOQoL-BREF captures an individual’s HRQoL across 4 independent domains: physical (7 items), psychological (6 items), social relationships (3 items), and environmental (8 items). Domain items, individually scored from 1 to 5, were summed and transformed into a 0-100 score, with higher scores indicating better HRQoL [23]. For *healthy* people, median (SD) domain scores were physical 76.5 (16.2), psychological 67.8 (15.6), social 70.5 (20.7), and environmental 68.2 (13.8) [24]. To the best of our knowledge, minimal clinically important differences for the WHOQoL-BREF have not been established for RA. Others have reported minimal clinically important differences of approximately 10% in WHOQoL-BREF domain scores [25].

### Ethical Approval

Approval was obtained in April 2017 from the National Research Ethics Service Committee North West—Liverpool Central Research Ethics Committee (reference: 17/NW/0217).

### Statistical Methods

Participants were eligible for this analysis if they provided written consent for their data to be analyzed, completed the baseline questionnaire, returned the actigraph, provided symptom reports on at least 50% of eligible days, completed ≥50% of the CSDs, and completed the WHOQoL-BREF on at least two of the three possible follow-up time points. Descriptive statistics summarizing demographics and baseline measures are presented as frequencies and medians with IQRs.

### Data Preparation

Within the study, data item collection frequencies differed: sleep and symptoms were measured daily, whereas HRQoL was measured at baseline and at approximately days 10, 20, and 30. To preserve temporal ordering, we examined the relationship between daily sleep and symptom data from baseline to day 10, from day 11 to 20, and from day 21 to 30, with HRQoL at days 10, 20, and 30.

**Figure 2:**
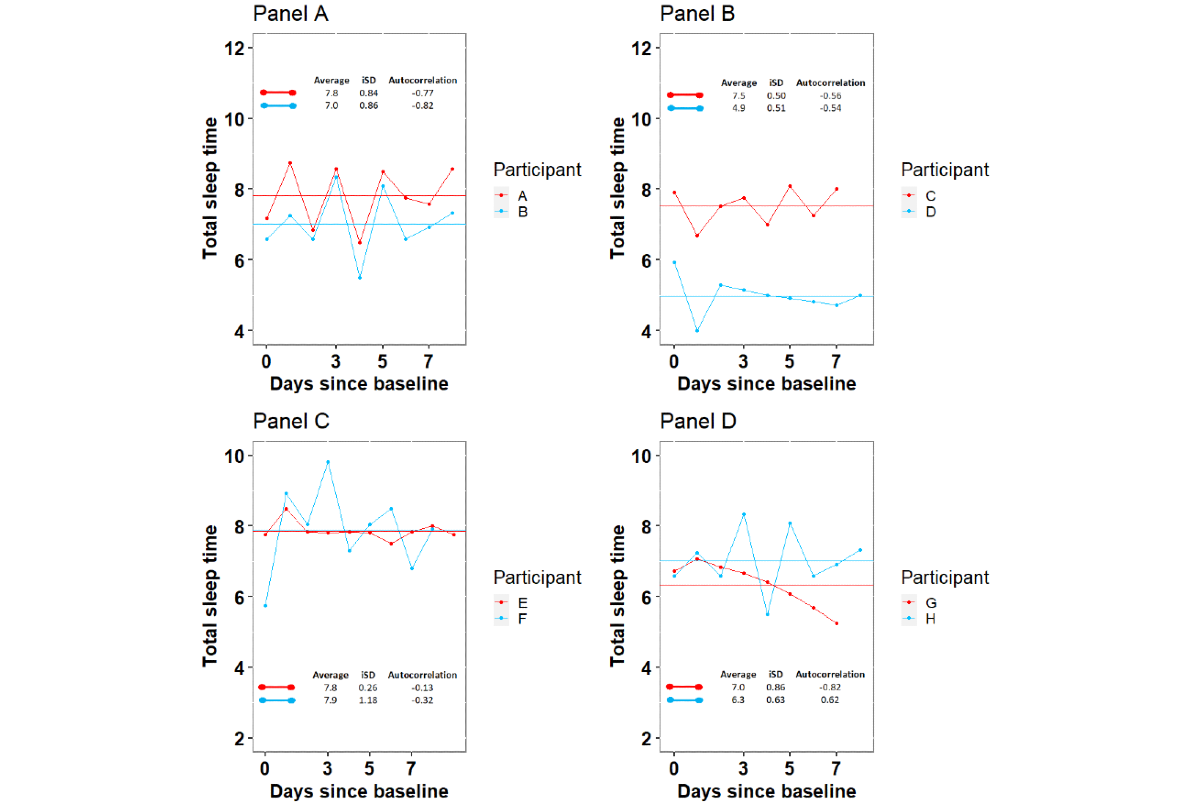
Examples of individual participants’ actigraphy assessed daily total sleep time showing average, intraindividual SD (iSD), and autocorrelation scores over 10 days. In this figure, each panel plots the daily total sleep time for 2 selected participants over 10 days. The 10-day average sleep time is shown as a straight line. In all, 2 measures of variability of total sleep time across the 10-day period were calculated, the iSD and the autocorrelation. The individual panels show the following: (A) shows 2 participants with similar average iSD and autocorrelation scores, (B) shows 2 participants with different average but similar iSD and autocorrelation scores, and (C) shows 2 participants with similar average and autocorrelation scores. The higher iSD score of participant F reflects the higher amplitude of fluctuations in total sleep time when compared with the low amplitude of fluctuation in the total sleep time of participant E. (D) shows 2 participants with similar average scores. The autocorrelation score of participant H toward −1 reflects the fluctuation in total sleep time, whereas the autocorrelation score of participant G toward 1 reflects the day-to-day stability in total sleep time despite a decrease over the period of observation.

For the sleep and symptom variables, we calculated the average score and 2 measures of score variability, intraindividual SD (iSD), and autocorrelation [26] (Figure 2). The *average* sleep and symptom scores were calculated as a simple arithmetic mean over each 10-day time window. *iSD* captures the amplitude of sleep or symptom score fluctuations, with higher values indicating higher amplitude and therefore increased between-day variability. *Autocorrelation* (temporal dependency) assesses the extent to which sleep or symptom scores can be predicted based on previous scores. Autocorrelation values ranged from −1 (indicating significant fluctuations around the mean value) to 1 (indicating stable scores at or above or below the mean value).

### Data Analysis

The data for this study were organized at 2 levels. The first level was time (within-person), which was nested within the second level, individuals (between-person). Thus, we used a multilevel data analysis strategy in all analyses. First, univariable multilevel models were fitted to investigate the association between the average sleep scores and each of the 4 WHOQoL-BREF domains. To avoid multicollinearity, separate models were constructed for each sleep variable. To estimate the direct effect of average sleep scores on HRQoL (denoted by the green arrow in Figure 1), multivariable models were fitted to adjust for baseline factors (age, sex, Index of Multiple Deprivation, smoking status, alcohol consumption, marital status, number of medications, BMI, Hospital Anxiety and Depression Scale—anxiety subscale, RAPID-3, PSQI, SCI, OSA, and RLS). The models were then adjusted for sleep variability (iSD and autocorrelation) and consecutively for pain, mood, and fatigue. Finally, all variables were entered into the model. The results are presented as *β*-coefficients with 95% CIs. The variance in the outcome explained by fixed (marginal *R*^2^) and combined fixed and random (conditional *R*^2^) effects was used to assess model performance.

All analyses were performed using R (version 3.6.0; R Foundation for Statistical Computing).

## Results

### Study Cohort

A total of 9428 emails were sent to the nonmembers and registered members of the National Rheumatoid Arthritis Society. In total, 285 participants were recruited for the study (Figure S2 in Multimedia Appendix 1). Of 285 participants, 268 (94%) provided baseline data, consent, and returned the actigraph; and 254 (89.1% of recruited participants, 94.8% of eligible participants) were included in the analysis. The 254 participants provided 6731 person-days of CSD data (88.3% of the maximum possible; N=7620) and 7299 person-days of symptom reports (95.8% of the maximum possible; N=7620). The baseline characteristics of the study cohort are presented in Table 1. Sleep problems were common, with a median PSQI score of 11 (IQR 10-12); 32.3% (82/254) of the participants had probable insomnia (SCI score ≤16), 5.9% (15/254) had OSA, and 9.4% (24/254) had RLS.

**Table 1:** Cohort characteristics.

Characteristics		Participants with baseline data (N=268)	Participants in analysis (n=254)
Sex (female), n (%)		219 (81.7)	206 (81.1)
Age^a^ (years), median (IQR)		57 (49-65)	57 (49-64)
**Marital status, n (%)**			
	Single	21 (7.8)	19 (7.5)
	Married or with partner	202 (75.4)	194 (76.4)
	Separated, widowed, or divorced	44 (16.4)	39 (15.3)
	Missing	1 (0.4)	2 (0.8)
Deprivation decile^a^ (1=most deprived, 10=least deprived), median (IQR)		7 (4-8)	7 (4-8)
Disease duration^a^ (years), median (IQR)		8.8 (3.6-13.9)	8.4 (3.34-13.8)
Baseline disease activity^a^ (Routine Assessment of Patient Index Data 3), median (IQR)		14.3 (8.3-19.4)	14.2 (8.3-19.3)
Number of medications^a^, median (IQR)		3 (2-4)	3 (2-4)
Possible insomnia (Sleep Condition Indicator; score ≤16), n (%)		83 (31)	82 (32.3)
Pittsburgh Sleep Quality Index^a^, median (IQR)		11 (10-12)	11 (10-12)
Sleep apnea (yes), n (%)		16 (6)	15 (5.9)
Restless leg syndrome (yes), n (%)		25 (9.3)	24 (9.4)
**Smoking, n (%)**			
	Current smoker	22 (8.2)	21 (8.3)
	Ex-smoker	106 (39.6)	99 (39)
	Never smoker	137 (51.1)	130 (51.2)
	Missing	3 (1.1)	4 (1.6)
**Alcohol^b^, n (%)**			
	None	108 (40.3)	102 (40.2)
	Moderate	138 (51.5)	130 (51.2)
	Heavy	21 (7.8)	21 (8.3)
	Missing	1 (0.4)	1 (0.4)
**BMI^c^ (kg/m^2^), n (%)**			
	Underweight	5 (1.9)	5 (2)
	Healthy	95 (35.5)	88 (34.6)
	Overweight	79 (29.5)	73 (28.7)
	Obese	81 (30.2)	79 (31.1)
	Missing	8 (3)	9 (3.5)
**Anxiety^d^ (Hospital Anxiety and Depression Scale—anxiety subscale), n (%)**			
	Not case (0 to <8)	56 (21)	52 (20.5)
	Borderline case (8 to <11)	146 (54.5)	138 (54.3)
	Case (11 to 21)	65 (24.3)	62 (24.4)
	Missing	1 (0.4)	2 (0.8)

^a^Missing values are not shown.

^b^None: 0 units; moderate: 1-15 units; and heavy: ≥16 units.

^c^Underweight: <18.5 kg/m^2^; healthy: 18.5-24.9 kg/m^2^; overweight: 24.9-30 kg/m^2^; or obese: >30 kg/m^2^.

^d^Not case: 0-8; borderline case: 8-11; and definite case: 11-21.

**Figure 3:**
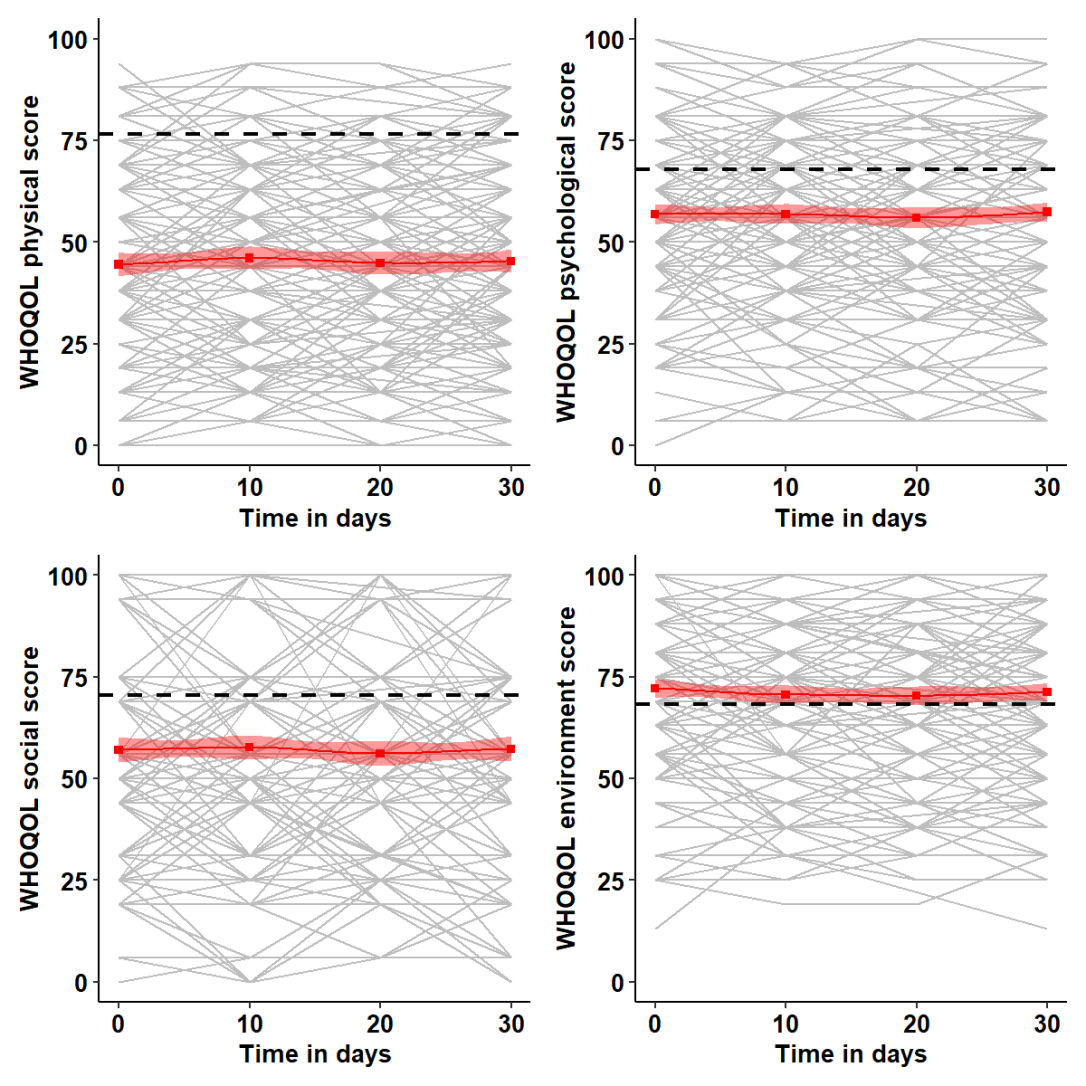
Plot of individual participant World Health Organization Quality of Life-Brief (WHOQoL-BREF) domain scores across 30 days. The blue line is the cohort mean score, the black dashed line is the mean score for healthy individuals.

Individual participant’s WHOQoL-BREF scores were plotted separately for each of the 4 domains (Figure 3). The cohort mean is shown in red, and the mean scores for healthy individuals are shown in black. At all time points, the mean scores across all participants were lower, that is, poorer, when compared with healthy individuals for the physical, psychological, and social HRQoL domains. The mean score for the environmental domain was similar for the RA cohort and general population. There was substantial variability in domain scores between individuals and change over time within individuals, with; for example, 20.1% (51/254) of the participants having a ≥10% decrease in the physical domain score between consecutive assessments.

**Figure 4:**
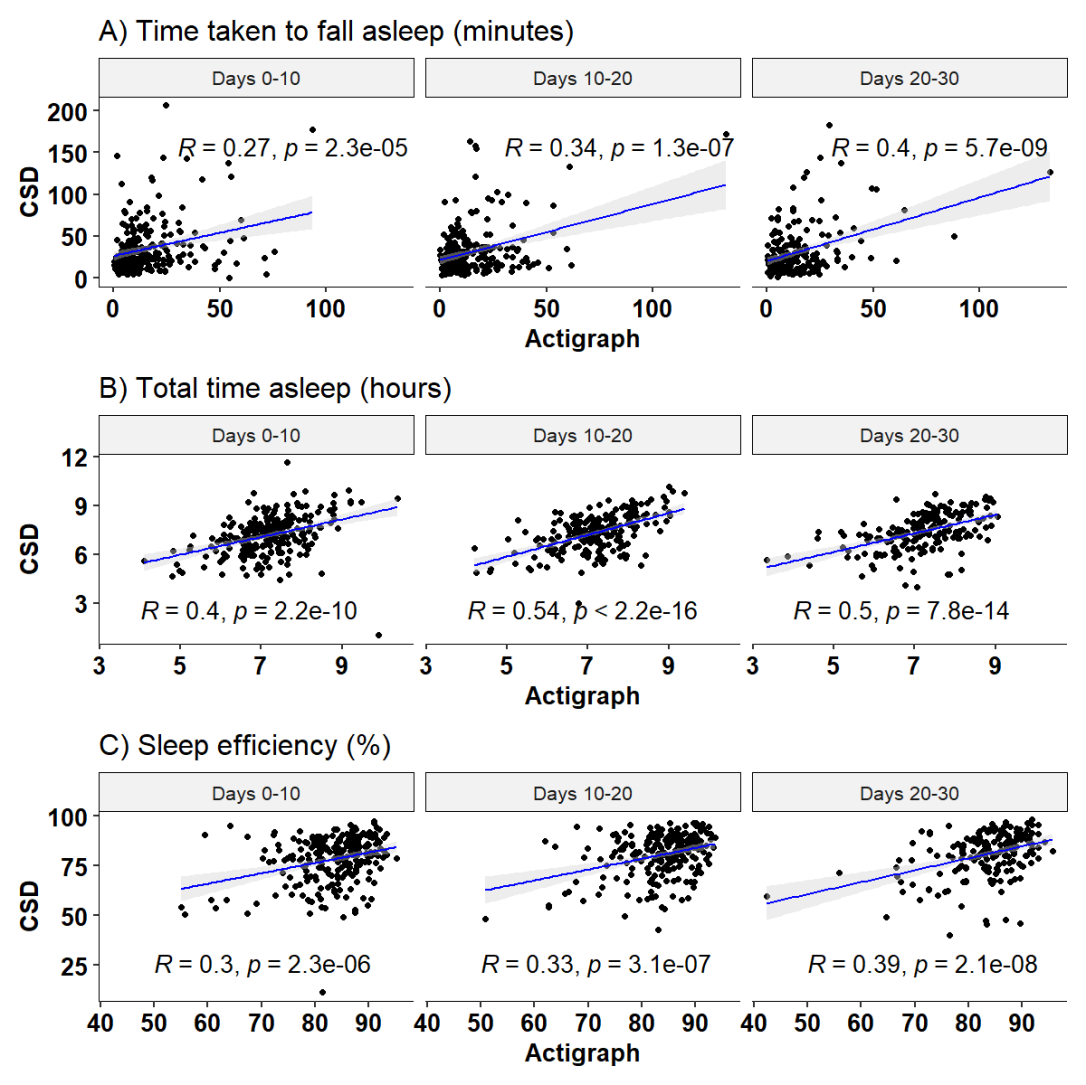
Correlation between objective (actigraph) and subjective (Consensus Sleep Diary [CSD]) measured time taken to fall asleep (A), total time asleep (B), and sleep efficiency (C).

Participants’ self-reported sleep patterns on the CSD did not correlate strongly with actigraph data. From baseline to day 10, the median (IQR) time taken to fall asleep was higher on the CSD (median 35.6, IQR 15.4-42.4 minutes) when compared with the actigraph (median 16.1, IQR 6.1-20.6 minutes; Table S1 in Multimedia Appendix 2), and the correlation between these 2 measures was weak (Pearson *r*=0.27; Figure 4). Similarly, low correlations were found for total time asleep (*r*=0.4) although the median total time asleep for both measures was 7.2 hours, and sleep efficiency (*r*=0.3) was lower on the CSD (median 78.0, IQR 71.9-86.9, cf. median 83.3, IQR 80.1-87.7). Participants reported poor sleep quality (median score 2.0, IQR 1.6-2.5) and did not feel refreshed on wakening (median score 1.6, IQR 1.1-2.1). The median actigraphy fragmentation index was 32.3 (IQR 22.0-38.6). These patterns were similar across days 11-20 and 21-30 (Figure 3; Table S1 in Multimedia Appendix 2).

### The Relationship Between Sleep and HRQoL and the Effect of Pain, Mood, and Fatigue

#### CSD Sleep Variables

##### Overview

The results of the multilevel models to examine the relationship between CSD sleep variables and the WHOQoL-BREF domains are shown in Table 2 and are summarized in the following sections.

**Table 2:** Consensus Sleep Diary and health-related quality of life.^a^

Sleep parameter and quality of life domain			Univariable *β* (95% CI)		Multivariable *β* (95% CI)								
					Baseline factors^b^		Baseline factors plus pain^c^		Baseline factors plus mood^c^		Baseline factors plus fatigue^c^		Baseline factors plus pain, mood, and fatigue^c^
**Time taken to fall asleep (minutes)^d^**													
	Environmental	−0.29 (−0.63 to 0.04)		−0.082 (−0.42 to 0.23)		0.14 (−0.34 to 0.59)		0.13 (−0.34 to 0.58)		0.10 (−0.36 to 0.54)		0.43 (−0.10 to 0.95)	
	Physical	−0.47 (−0.86 to −0.08)^e^		−0.41 (−0.78 to −0.09)^e^		−0.47 (−0.97 to −0.01)^e^		−0.19 (−0.68 to 0.25)		−0.41 (−0.88 to 0.03)		0.02 (−0.49 to 0.51)	
	Psychological	−0.71 (−1.07 to −0.35)^e^		−0.55 (−0.91 to −0.22)^e^		−0.47 (−0.99 to 0.01)		−0.22 (−0.72 to 0.24)		−0.25 (−0.73 to 0.22)		0.13 (−0.40 to 0.66)	
	Social	−0.75 (−1.29 to −0.21)^e^		−0.53 (−1.06 to −0.02)^e^		−0.60 (−1.34 to 0.12)		−0.70 (−1.44 to 0.04)		−0.65 (−1.38 to 0.07)		−0.26 (−1.06 to 0.57)	
**Total time asleep (hours)^f^**													
	Environmental	0.49 (−0.41 to 1.40)		0.36 (−0.48 to 1.27)		−0.09 (−1.11 to 1.00)		0.33 (−0.67 to 1.39)		0.03 (−0.92 to 1.02)		0.23 (−0.96 to 1.46)	
	Physical	1.11 (0.07 to 2.15)^e^		0.93 (0.04 to 1.82)^e^		0.39 (−0.64 to 1.42)		0.47 (−0.52 to 1.47)		0.45 (−0.50 to 1.41)		0.14 (−0.94 to 1.22)	
	Psychological	0.45 (−0.52 to 1.43)		0.41 (−0.49 to 1.35)		−0.07 (−1.16 to 1.08)		0.28 (−0.74 to 1.36)		0.01 (−0.99 to 1.04)		0.30 (−0.84 to 1.52)	
	Social	1.65 (0.21 to 3.10)^e^		1.50 (0.18 to 2.93)^e^		0.012 (−1.55 to 1.68)		0.88 (−0.68 to 2.57)		1.001 (−0.49 to 2.57)		−0.03 (−1.77 to 1.84)	
**Sleep efficiency (%)^g^**													
	Environmental	1.10 (0.14 to 2.06)^e^		0.48 (−0.426 to 1.45)		−0.42 (−1.73 to 0.99)		−0.12 (−1.41 to 1.24)		−0.05 (−1.29 to 1.25)		−0.74 (−2.25 to 0.82)	
	Physical	2.92 (1.81 to 4.05)^e^		2.03 (1.10 to 3.02)^e^		1.22 (−0.08 to 2.60)		0.98 (−0.28 to 2.32)		1.42 (0.19 to 2.71)^e^		0.01 (−1.36 to 1.43)	
	Psychological	2.44 (1.41 to 3.49)^e^		1.80 (0.85 to 2.82)^e^		2.11 (0.71 to 3.61)^e^		1.57 (0.24 to 2.98)^e^		1.67 (0.38 to 3.04)^e^		1.28 (−0.20 to 2.84)	
	Social	2.97 (1.44 to 4.50)^e^		2.22 (0.78 to 3.76)^e^		1.53 (−0.50 to 3.72)		1.92 (−0.12 to 4.09)		2.02 (0.05 to 4.10)^e^		0.823 (−1.45 to 3.20)	
**Sleep quality (1-5)**													
	Environmental	1.65 (0.20 to 3.12)^e^		−0.05 (−1.46 to 1.47)		0.31 (−1.46 to 2.23)		0.03 (−1.82 to 2.01)		0.12 (−1.59 to 1.90)		0.10 (−2.21 to 2.54)	
	Physical	5.99 (4.34 to 7.69)^e^		4.58 (3.12 to 6.11)^e^		4.06 (2.29 to 5.91)^e^		3.97 (2.16 to 5.81)^e^		3.83 (2.11 to 5.61)^e^		3.13 (1.03 to 5.31)^e^	
	Psychological	2.47 (0.89 to 4.10)^e^		0.95 (−0.55 to 2.58)		1.06 (−0.84 to 3.17)		1.10 (−0.78 to 3.20)		1.12 (−0.68 to 3.09)		1.54 (−0.71 to 4.01)	
	Social	2.82 (0.44 to 5.24)^e^		0.04 (−2.25 to 2.56)		−0.25 (−2.97 to 2.69)		−0.22 (−3.15 to 3.00)		0.21 (−2.55 to 3.15)		−1.03 (−4.46 to 2.68)	
**Feeling refreshed (1-5)**													
	Environmental	2.12 (0.63 to 3.65)^e^		0.49 (−0.90 to 2.07)		0.22 (−1.48 to 2.15)		−0.05 (−1.80 to 1.88)		−0.38 (−2.09 to 1.44)		−0.85 (−3.07 to 1.56)	
	Physical	6.28 (4.57 to 8.04)^e^		4.57 (3.14 to 6.10)^e^		3.99 (2.31 to 5.76)^e^		4.31 (2.59 to 6.06)^e^		3.44 (1.72 to 5.22)^e^		3.60 (1.58 to 5.65)^e^	
	Psychological	3.73 (2.07 to 5.44)^e^		2.43 (0.98 to 4.15)^e^		2.25 (0.47 to 4.35)^e^		2.13 (0.37 to 4.16)^e^		1.94 (0.18 to 3.93)^e^		1.87 (−0.32 to 4.34)	
	Social	2.97 (0.5 to 5.42)^e^		0.74 (−1.49 to 3.31)		−0.62 (−3.23 to 2.39)		−0.49 (−3.26 to 2.60)		−0.30 (−3.05 to 2.71)		−1.42 (−4.71 to 2.29)	

^a^The relationship between average scores of Consensus Sleep Diary and health-related quality of life.

^b^Age, sex, Index of Multiple Deprivation, smoking status, alcohol consumption, marital status, number of medications, BMI (self-reported kg/m^2^), Hospital Anxiety and Depression Scale—anxiety subscale, Routine Assessment of Patient Index Data 3, obstructive sleep apnea, and restless leg syndrome.

^c^Similar to footnote *b*, plus intraindividual SD and autocorrelation measures of sleep parameters, pain, mood, and fatigue.

^d^For each 10-minute increase in time taken to fall asleep.

^e^The results excluding zero.

^f^For each 1-hour asleep.

^g^For each 10% increase in sleep efficiency.

##### Time Taken to Fall Asleep

In the unadjusted models, an increase in the time taken to fall asleep was associated with lower, that is, poorer WHOQoL-BREF scores. For each 10-minute increase in the time taken to fall asleep, physical domain scores decreased by 0.47 points (*β*=−.47, 95% CI −0.86 to −0.08), psychological domain scores decreased by 0.71 points (95% CI −1.07 to −0.35), and social domain scores decreased by 0.75 points (95% CI −1.29 to −0.21). These associations persisted after adjusting for baseline factors (age, sex, deprivation, smoking status, alcohol consumption, marital status, number of medications used, BMI, and anxiety), baseline RAPID-3 scores, baseline PSQI and SCI scores, OSA, and RLS. When pain was included in the model, the relationship with physical domain scores was attenuated but persisted (*β*=−.05, 95% CI −0.10 to −0.001), whereas the relationship with psychological and social domain scores did not persist. When pain, mood, and fatigue were included in the final model, the time taken to fall asleep was not significantly associated with any of the WHOQoL-BREF domains (Table 2). The variability in time taken to fall asleep was not associated with the WHOQoL-BREF domains (Table S2 in Multimedia Appendix 2).

##### Total Time Asleep

An increase in the total time asleep was associated with higher, that is, better, physical (for each 1-hour increase: *β*=1.11, 95% CI 0.07-2.15) and social domain (*β*=1.65, 95% CI 0.21-3.10) scores. These associations were independent of baseline factors but were attenuated and no longer significant when pain, fatigue, and mood were included in the models. Variability in total time asleep was an important predictor of WHOQoL-BREF domains (Table S2 in Multimedia Appendix 2). An increase in the iSD score, indicating increased variability in the total sleep time, was associated with poorer physical (for each unit increase: *β*=−2.41, 95% CI −4.14 to −0.76) and psychological (*β*=−2.21, 95% CI −3.96 to −0.38) domain scores. These associations were not explained by the inclusion of baseline factors, such as pain, fatigue, and mood, into the models.

##### Sleep Efficiency

After adjusting for baseline factors, increased sleep efficiency was associated with better physical (for each 10% increase in sleep efficiency: *β*=2.03, 95% CI 1.10-3.02), psychological (*β*=1.80, 95% CI 0.85-2.82), and social domain scores (*β*=2.22, 95% CI 0.78-3.76). However, when pain, fatigue, and mood were included in the models, there were no significant associations between the sleep efficiency and HRQoL domains. The variability in sleep efficiency was not associated with the WHOQoL-BREF domains (Table S2 in Multimedia Appendix 2).

##### Sleep Quality

Sleep quality was associated with all 4 WHOQoL-BREF domains (Table 2). Although most associations were explained by baseline factors, the association between sleep quality and the physical domain scores persisted, with a unit increase in the sleep quality score being associated with a 4.58 (95% CI 3.12-6.11) increase in the physical domain score. This association was not explained by pain, mood, or fatigue. The variability in sleep quality was not associated with the WHOQoL-BREF domains (Table S2 in Multimedia Appendix 2).

##### Feeling Refreshed

A unit increase in the feeling refreshed score was associated with better physical (*β*=4.57, 95% CI 3.14-6.10) and psychological (*β*=2.43, 95% CI 0.98-4.15) HRQoL independently of baseline factors, including disease severity. The relationship with physical HRQoL persisted when pain, mood, and fatigue were included in the model. Variability in feeling refreshed was not associated with WHOQoL-BREF domains (Table S2 in Multimedia Appendix 2).

#### Actigraphy Sleep Variables

Of the actigraph sleep parameters, only total sleep time was associated with physical domain scores and appeared in the opposite direction (*β*=−1.24, 95% CI −2.58 to −0.09; Table S3 in Multimedia Appendix 2). However, this association was attenuated and not significant when adjusted for pain, mood, and fatigue.

### Model Performance

The marginal *R*^2^ values of the final multivariable models ranged from 33% to 69% (Multimedia Appendix 2).

## Discussion

### Principal Findings

In this study, we report the findings of a prospective mHealth study that examined the role of sleep disturbance in people with RA and its impact on HRQoL. At all assessment points, the average HRQoL of the cohort was lower than the population average [24]. We observed consistent patterns of association between sleep and HRQoL. First, the CSD sleep variables predicted physical, psychological, and social HRQoL: increases in the time taken to fall asleep predicted poorer HRQoL, whereas increases in total time asleep, sleep efficiency, feeling refreshed, and sleep quality predicted better HRQoL. Increased variability in total sleep time was associated with poor physical and psychological quality of life. Second, most of these associations were independent of sociodemographic and lifestyle factors; disease activity; baseline medication use; levels of anxiety; sleep quality (PSQI score); and clinical sleep disorders, including insomnia, OSA, and RLS. Third, these data clearly show that the relationship between sleep variables and HRQoL (with the exception of feeling refreshed, sleep quality with physical HRQoL, and the relationships with variability in total sleep time) were mediated via changes in pain, mood, and fatigue. Finally, there was no consistent pattern of association between actigraphy-derived sleep variables and HRQoL.

### Limitations

There are several limitations to consider when interpreting these results. First, our study design may have introduced a selection bias. For example, older age and higher disease severity have been shown to predict nonparticipation in digital health research [27]. Bias may have been introduced if nonparticipation was related to sleep disturbance and, independently of sleep, to HRQoL. However, our data indicate that this was unlikely to be the case because the rates of sleep disturbance were within the expected range. The median PSQI score was 11, which is comparable with that in other studies of people with RA [28]. The prevalence of poor sleep (PSQI score ≥5) was 90% (data not shown), which is in line with estimates from previous studies. Finally, one-third of our sample was classified as having probable insomnia, which is comparable with other estimates of patients with *arthritis* [29]. Our screening for OSA and RLS was limited, and their impact on RA-HRQoL was unclear in our study. Engagement in the study was high (241/254, 94.8%) among those participants who were recruited and successfully commenced data collection, and a few (14/268, 5.5%) were lost to follow-up after enrolling in the study. Therefore, it is unlikely that the loss to follow-up bias had a substantial influence on our results. Finally, we observed a poor correlation between the subjective (CSD) and objective (actigraphy) measures of sleep. The low correlation between subjective and objective measures is a common observation. They appear to measure different dimensions of sleep: the correlation between polysomnography measures of sleep and actigraphy is stronger than that between polysomnography and sleep diaries [30]. There may be different underlying biological (eg, inflammatory) and psychological mechanisms between subjective and objective measures, or self-reported sleep may reflect the reporting of a more chronic sleep problem, whereas actigraphy assesses acute sleep changes [30].

### Implications of This Study

The data reported here support our hypothesis that sleep disturbances predict poor HRQoL in people with RA. Sleep efficiency was low compared with healthy people [31], and people with RA spent substantial periods in bed but not asleep. Optimizing total sleep time, increasing sleep efficiency, decreasing sleep onset latency, and reducing variability in total sleep time could improve HRQoL in people with RA. We also reported that pain, mood, and fatigue mediate these relationships. There is no high-quality evidence for the effectiveness of pharmacological [32] or nonpharmacological [33] sleep interventions in people with RA. The hybrid cognitive behavioral therapy (CBT) proposed by Tang et al [34] for people with chronic pain incorporates components of CBT for insomnia and CBT for pain and has been shown to improve sleep, pain interference, fatigue, and depression. Our data suggest that hybrid treatment models that simultaneously address sleep disturbances and associated symptoms, including pain, mood, and fatigue, may improve HRQoL in people with RA.

### Conclusions

Sleep predicts poor HRQoL independent of disease severity. Sleep disturbance indirectly impacts poor HRQoL via its effects on pain, mood, and fatigue. These data should inform the development of complex interventions to improve sleep-related HRQoL in people with RA.

## Appendix

Multimedia Appendix 1 A figure of the main graphical interface used in the study and the flow chart of participants recruited and included in the study.

Multimedia Appendix 2 Tables summarizing the Consensus Sleep Diary and Actigraph derived sleep parameters, and the relationships between sleep parameters and health-related quality of life.

## Abbreviations

CBT: cognitive behavioral therapy
CSD: Consensus Sleep Diary
HRQoL: health-related quality of life
iSD: intraindividual SD
mHealth: mobile health
NHS: National Health Service
OSA: obstructive sleep apnea
PSQI: Pittsburgh Sleep Quality Index
QUASAR: quality of life, sleep, and rheumatoid arthritis
RA: rheumatoid arthritis
RAPID-3: Routine Assessment of Patient Index Data 3
RLS: restless leg syndrome
SCI: Sleep Condition Indicator
WHOQoL-BREF: World Health Organization Quality of Life-Brief
