# Supplementary material for: Sleep Disturbance and Quality of Life in Rheumatoid Arthritis: Prospective mHealth Study
Source: J Med Internet Res. 2022 Apr 22;24(4):e32825. doi: 10.2196/32825 (PMC9077504; doi:10.2196/32825)
Supplement: Multimedia Appendix 1 [file jmir_v24i4e32825_app1.docx]

**SUPPLEMENTARY MATERIALS**

**Figure S1. Main graphical interface used in the study. Each of the 10 segments represents a different symptom, such as pain severity (highlighted), measured on a 5-point ordinal scale.**

**
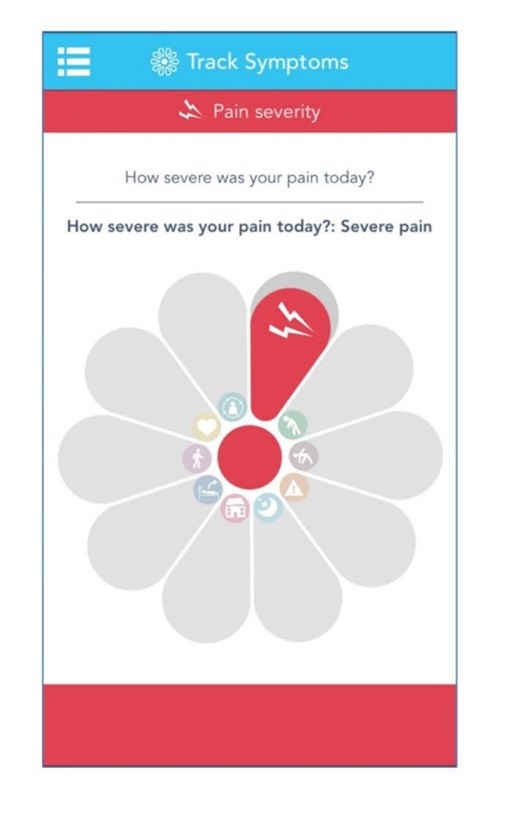
**

**Figure S2. Flow chart of participants recruited and included in the study.**

**
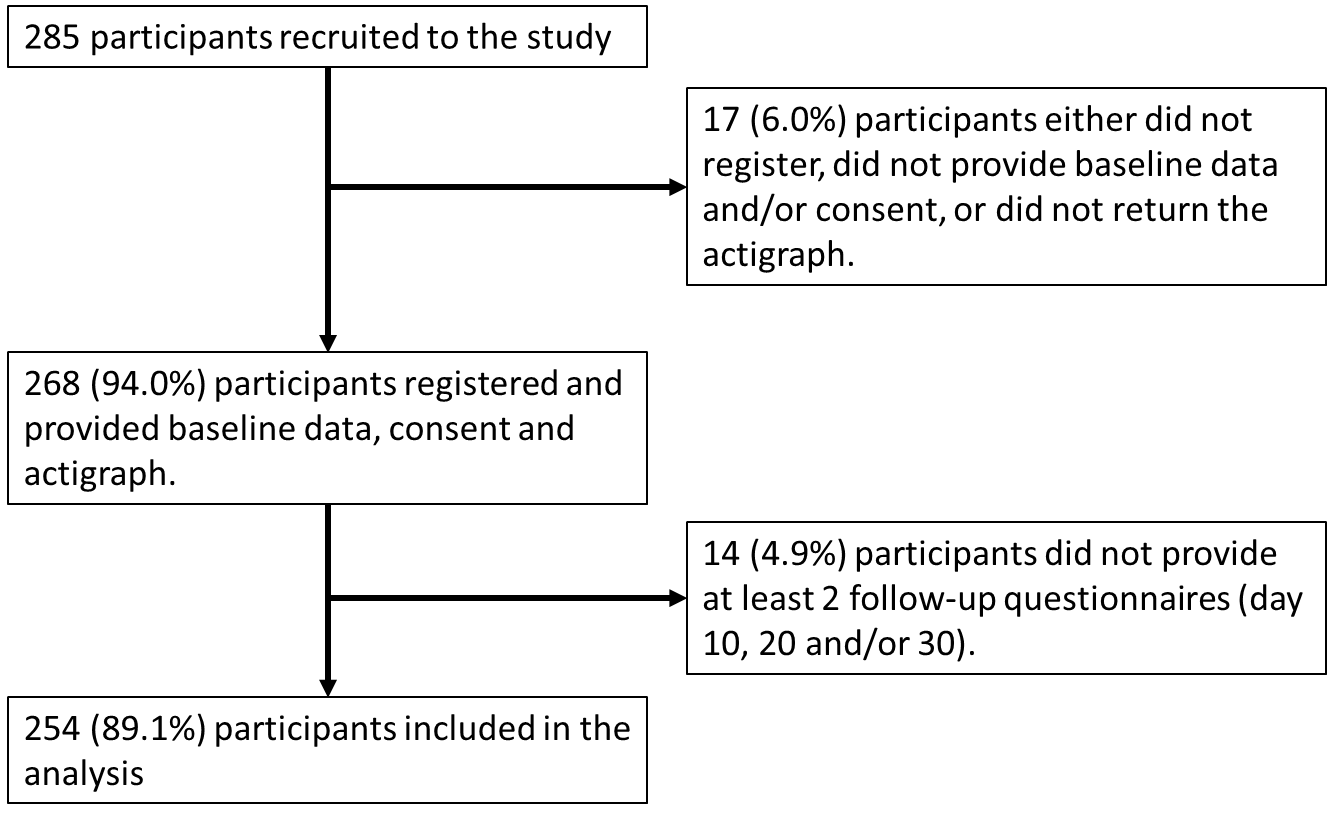
**
