# Supplementary material for: Sleep Disturbance and Quality of Life in Rheumatoid Arthritis: Prospective mHealth Study
Source: J Med Internet Res. 2022 Apr 22;24(4):e32825. doi: 10.2196/32825 (PMC9077504; doi:10.2196/32825)
Supplement: Multimedia Appendix 2 [file jmir_v24i4e32825_app2.docx]

| **Table S1. Summary of Consensus Sleep Diary and Actigraph derived sleep parameters** | | | | | | | | | |
| --- | --- | --- | --- | --- | --- | --- | --- | --- | --- |
| Sleep parameter | **Day 0-10** | | | **Day 11-20** | | | **Day 21-30** | | |
|  | Average | iSD | Autocorr. | Average | iSD | Autocorr. | Average | iSD | Autocorr. |
| **Consensus Sleep Diary** | | | | | | | | | |
| Time taken to fall asleep (minutes) | 35.6  (15.4-42.4) | 27.7  (8.8-37.8) | -0.00  (-0.21-0.21) | 31.5  (13.6-37.4) | 24.2  (7.3-30.6) | -0.01  (-0.20-0.17) | 31.9  (11.7-39.2) | 21.7  (5.6-31.9) | -0.00  (-0.19-0.18) |
| Total time asleep (hours) | 7.2  (6.5-7.9) | 1.2  (0.8-1.6) | -0.07  (-0.3-0.2) | 7.3  (6.6-8.1) | 1.1  (0.8-1.4) | -0.03  (-0.3-0.2) | 7.4  (6.8-8.2) | 1.1  (0.8-1.3) | 0  (-0.2-0.2) |
| Sleep efficiency (%) | 78.0  (71.9-86.9) | 10.8  (6.2-13.9) | -0.03  (-0.3-0.2) | 80.34  (74.7-88.2) | 9.5  (5.2-11.8) | -0.05  (-0.2-0.1) | 80.6  (74.5-88.4) | 8.9  (5.0-11.4) | 0  (-0.2-0.2) |
| Sleep quality  (1-5) | 2.0  (1.6-2.5) | 0.8  (0.6-1.0) | -0.07  (-0.3-0.1) | 2.1  (1.7-2.7) | 0.7  (0.5-0.9) | -0.07  (-0.3-0.1) | 2.2  (1.7-2.6) | 0.7  (0.5-0.9) | -0.1  (-0.2-0.1) |
| Feeling refreshed (1-5) | 1.6  (1.1-2.1) | 0.7  (0.6-0.9) | -0.06  (-0.3-0.1) | 1.7  (1.2-2.2) | 0.7  (0.5-0.9) | -0.08  (-0.3-0.1) | 1.8  (1.3-2.2) | 0.7  (0.5-0.9) | -0.1  (-0.3-0.1) |
|  |  |  |  |  |  |  |  |  |  |
| **Actigraph** | | | | | | | | | |
| Time taken to fall asleep (minutes) | 16.1  (6.1-20.6) | 15.5  (6.9-19.5) | -0.08  (-0.27-0.10) | 15.3 (5.6-21.3) | 15.2 (6.9-20.9) | -0.07 (-0.25-0.06) | 14.9  (5.2-18.8) | 14.5  (6.6-19.6) | -0.01  (-0.21-0.16) |
| Total time asleep (hours) | 7.2  (6.7-7.7) | 0.9  (0.6-1.0) | -0.04  (-0.27-0.17) | 7.2  (6.7-7.8) | 0.9  (0.6-1.0) | -0.04  (-0.3-0.2) | 7.2  (6.7-7.8 | 0.8  (0.5-1.0) | 0  (-0.2-0.1) |
| Sleep efficiency (%) | 83.3  (80.1,87.7) | 4.4  (2.6-5.5) | -0.09  (-0.28-0.11) | 83.4  (81.1-87.8) | 4.3  (2.9-5.1) | -0.10  (-0.3-0.1) | 83.3  (80.1-87.9) | 4.5  (2.9-5.3) | -0.1  (-0.3-0.1) |
| Fragmentation Index | 32.3  (22.0,38.6) | 9.6  (6.3-11.5) | -0.08  (-0.32-0.16) | 32.4  (23.0-38.0) | 9.5  (6.6-11.6) | -0.07  (-0.3-0.1) | 31.6  (23.1-37.3) | 9.4  (6.5-11.6) | -0.1  (-0.3-0.1) |
| All values are median (inter quartile range)  iSD = intra-individual Standard Deviation. Autocorr. = autocorrelation | | | | | | | | | |

| **Table S2. The relationship between Consensus Sleep Diary and WHOQoL domains** | | | | | | | | | |
| --- | --- | --- | --- | --- | --- | --- | --- | --- | --- |
| **Sleep parameter** | **Quality of life domain** | **Sleep parameter measure** | **Univariable** | **Multivariable** | | **R^2^ univariable** | | **R^2^ multivariable (all covariates)** | |
|  |  |  |  | Baseline factors^1^ | All co-variates^3^ | Marginal | Conditional | Marginal | Conditional |
| **Time taken to fall asleep**  **(Minutes)** | Environment | Average | -0.01 (-0.04,0.02) | -0.01 (-0.04,0.02) | -0.01 (-0.04,0.02) | 0.00 | 0.89 | 0.40 | 0.88 |
|  |  | iSD |  |  | -0.01 (-0.04,0.02) |  |  |  |  |
|  |  | Autocorrelation |  |  | -0.01 (-0.04,0.02) |  |  |  |  |
|  | Physical | Average | -0.05 (-0.09,-0.01) | -0.04 (-0.08,-0.01) | 0 (-0.05,0.05) | 0.00 | 0.91 | 0.66 | 0.91 |
|  |  | iSD |  |  | -0.01 (-0.06,0.04) |  |  |  |  |
|  |  | Autocorrelation |  |  | 1.08 (-1.96,4.03) |  |  |  |  |
|  | Psychological | Average | -0.07 (-0.11,-0.03) | -0.06 (-0.09,-0.02) | 0.01 (-0.04,0.07) | 0.01 | 0.9 | 0.52 | 0.88 |
|  |  | iSD |  |  | -0.05 (-0.1,0) |  |  |  |  |
|  |  | Autocorrelation |  |  | 2.72 (-0.51,5.79) |  |  |  |  |
|  | Social | Average | -0.07 (-0.13,-0.02) | -0.05 (-0.11,0) | -0.03 (-0.11,0.06) | 0.01 | 0.82 | 0.32 | 0.82 |
|  |  | iSD |  |  | 0.02 (-0.06,0.1) |  |  |  |  |
|  |  | Autocorrelation |  |  | 0.37 (-4.64,5.34) |  |  |  |  |
| **Sleep efficiency**  **(%)** | Environment | Average | **0.11 (0.01,0.21)** | 0.05 (-0.04,0.14) | -0.07 (-0.23,0.08) | 0.00 | 0.89 | 0.40 | 0.87 |
|  |  | iSD |  |  | -0.13 (-0.33,0.08) |  |  |  |  |
|  |  | Autocorrelation |  |  | -0.44 (-3.73,2.58) |  |  |  |  |
|  | Physical | Average | **0.29 (0.18,0.40)** | **0.2 (0.11,0.30)** | 0 (-0.14,0.14) | 0.02 | 0.91 | 0.66 | 0.91 |
|  |  | iSD |  |  | **-**0.21 (-0.4,0.02) |  |  |  |  |
|  |  | Autocorrelation |  |  | 0.6 (-2.4,3.46) |  |  |  |  |
|  | Psychological | Average | **0.24 (0.14,0.35)** | **0.18 (0.08,0.28)** | 0.13 (-0.02,0.28) | 0.02 | 0.9 | 0.53 | 0.88 |
|  |  | iSD |  |  | -0.03 (-0.23,0.18) |  |  |  |  |
|  |  | Autocorrelation |  |  | -1.37 (-4.58,1.71) |  |  |  |  |
|  | Social | Average | **0.30 (0.14,0.45)** | **0.22 (0.08,0.38)** | 0.08 (-0.15,0.32) | 0.02 | 0.81 | 0.32 | 0.82 |
|  |  | iSD |  |  | 0 (-0.31,0.32) |  |  |  |  |
|  |  | Autocorrelation |  |  | -0.05 (-4.92,4.74) |  |  |  |  |
| **Total time asleep**  **(Hour)** | Environment | Average | 0.49 (-0.41,1.4) | 0.36 (-0.48,1.27) | 0.23 (-0.96,1.46) | 0 | 0.89 | 0.4 | 0.87 |
|  |  | iSD |  |  | -0.98 (-2.75,0.83) |  |  |  |  |
|  |  | Autocorrelation |  |  | 0.16 (-2.63,2.96) |  |  |  |  |
|  | Physical | Average | **1.11 (0.07,2.15)** | **0.93 (0.04,1.82)** | 0.14 (-0.94,1.21) | 0.00 | 0.91 | 0.67 | 0.91 |
|  |  | iSD |  |  | **-2.41 (-4.14,-0.76)** |  |  |  |  |
|  |  | Autocorrelation |  |  | -0.16 (-2.81,2.51) |  |  |  |  |
|  | Psychological | Average | 0.45 (-0.52,1.43) | 0.41 (-0.49,1.35) | 0.3 (-0.84,1.52) | 0.00 | 0.9 | 0.52 | 0.88 |
|  |  | iSD |  |  | **-2.21 (-3.96,-0.38)** |  |  |  |  |
|  |  | Autocorrelation |  |  | -0.34 (-3.17,2.43) |  |  |  |  |
|  | Social | Average | **1.65 (0.21,3.1)** | **1.5 (0.18,2.92)** | -0.03 (-1.77,1.84) | 0.01 | 0.82 | 0.32 | 0.82 |
|  |  | iSD |  |  | -2.58 (-5.33,0.2) |  |  |  |  |
|  |  | Autocorrelation |  |  | -1.45 (-5.84,2.94) |  |  |  |  |
| **Sleep quality**  **(1-5)** | Environment | Average | **1.65 (0.2,3.12)** | -0.05 (-1.46,1.47) | 0.10 (-2.21,2.54) | 0.00 | 0.89 | 0.4 | 0.87 |
|  |  | iSD |  |  | 1.82 (-2.16,6.09) |  |  |  |  |
|  |  | Autocorrelation |  |  | 2.56 (-1.24,6.28) |  |  |  |  |
|  | Physical | Average | **5.99 (4.34,7.69)** | **4.58 (3.12,6.11)** | **3.13 (1.03,5.31)** | 0.04 | 0.9 | 0.67 | 0.9 |
|  |  | iSD |  |  | -2.77 (-6.58,0.99) |  |  |  |  |
|  |  | Autocorrelation |  |  | 0.67 (-2.83,4.23) |  |  |  |  |
|  | Psychological | Average | **2.47 (0.89,4.1)** | 0.95 (-0.55,2.58) | 1.54 (-0.71,4.01) | 0.01 | 0.89 | 0.52 | 0.87 |
|  |  | iSD |  |  | -1.44 (-5.49,2.69) |  |  |  |  |
|  |  | Autocorrelation |  |  | -2.55 (-6.39,1.21) |  |  |  |  |
|  | Social | Average | **2.82 (0.44,5.24)** | 0.04 (-2.25,2.56) | -1.03 (-4.46,2.68) | 0.01 | 0.81 | 0.33 | 0.82 |
|  |  | iSD |  |  | -1.38 (-7.99,4.96) |  |  |  |  |
|  |  | Autocorrelation |  |  | -3.31 (-9.12,2.52) |  |  |  |  |
| **Feeling refreshed**  **(1-5)** | Environment | Average | **6.28 (4.57,8.04)** | **4.57 (3.14,6.1)** | **3.6 (1.58,5.65)** | 0.05 | 0.90 | 0.67 | 0.90 |
|  |  | iSD |  |  | -2.96 (-7.2,1.32) |  |  |  |  |
|  |  | Autocorrelation |  |  | 1.18 (-2.29,4.71) |  |  |  |  |
|  | Physical | Average | **3.73 (2.07,5.44)** | **2.43 (0.98,4.15)** | 1.87 (-0.32,4.34) | 0.02 | 0.89 | 0.51 | 0.86 |
|  |  | iSD |  |  | -1.94 (-6.62,2.83) |  |  |  |  |
|  |  | Autocorrelation |  |  | 0.76 (-3.04,4.74) |  |  |  |  |
|  | Psychological | Average | **2.96 (0.55,5.42)** | 0.74 (-1.48,3.31) | -1.42 (-4.71,2.29) | 0.01 | 0.81 | 0.31 | 0.82 |
|  |  | iSD |  |  | -2.35 (-9.47,4.81) |  |  |  |  |
|  |  | Autocorrelation |  |  | -3.09 (-8.86,2.86) |  |  |  |  |
|  | Social | Average | **2.12 (0.63,3.65)** | 0.49 (-0.9,2.07) | -0.85 (-3.07,1.56) | 0.01 | 0.88 | 0.39 | 0.87 |
|  |  | iSD |  |  | 0.93 (-3.64,5.72) |  |  |  |  |
|  |  | Autocorrelation |  |  | 1.13 (-2.58,4.98) |  |  |  |  |
| WHOQoL-BREF - World Health Organization Quality of Life Brief  All results are β (95% confidence interval). Results which exclude zero are highlighted in bold.  iSD = intra-individual Standard Deviation. Higher values indicate higher amplitude of fluctuations.  Autocorrelation (temporal dependency) assesses the extent to which observations can be predicted from previous observations. Positive values up to 1 indicate stable predictor variable reports (above or below mean value) with little variation with higher values indicating increased stability. Values of 0 indicate low predictive ability. Negative values up to -1 indicate fluctuating predictor variable reports (i.e. if the value of the predictor variable is below the mean, the next value is likely to be above the mean; if the value of the predictor variable is above the mean the next value is likely to be below the mean) with lower values indicating increased fluctuations.  1 Age, sex, Index of Multiple Deprivation, smoking, alcohol, marital status, number of medications, body mass index (self-reported kg/m2), Hospital Anxiety and Depression anxiety scale, RAPID-3, Obstructive Sleep Apnea, and Restless Leg Syndrome  2 As (1) above plus sleep parameter iSD and Autocorrelation  3 As (2) above plus average, iSD and autocorrelation measures of pain, mood and fatigue | | | | | | | | | |

| **Table S3. Actigraphy sleep parameters* and Health Related Quality of Life** | | | | | | | |
| --- | --- | --- | --- | --- | --- | --- | --- |
| **Sleep parameter** | **Quality of life domain** | **Univariable** | **Multivariable** | | | | |
|  |  |  | Baseline factors^1^ | Baseline factors plus pain^2^ | Baseline factors plus mood^2^ | Baseline factors plus fatigue^2^ | Baseline factors plus pain, mood and fatigue^2^ |
| **Time taken to fall asleep (minutes)** | Environment | -0.060 (-0.128, 0.008) | -0.001 (-0.070, 0.065) | 0.102 (-0.017, 0.219) | 0.033 (-0.082, 0.151) | 0.049 (-0.050, 0.148) | 0.031 (-0.099, 0.165) |
|  | Physical | -0.072 (-0.149, 0.005) | -0.019 (-0.089, 0.051) | -0.038 (-0.157, 0.084) | -0.058 (-0.171, 0.065) | -0.074 (-0.174, 0.031) | -0.098 (-0.220, 0.029) |
|  | Psychological | -0.048 (-0.121, 0.025) | -0.014 (-0.088, 0.055) | -0.157 (-0.284, 0.034) | -0.166 (-0.284, -0.046) | -0.091 (-0.199, 0.012) | -0.199 (-0.330, -0.071) |
|  | Social | -0.065 (-0.173, 0.043) | 0.015 (-0.093, 0.122) | 0.009 (-0.176, 0.197) | 0.039 (-0.145, 0.229) | 0.044 (-0.116, 0.208) | 0.003 (-0.195, 0.206) |
| **Total time asleep**  **(Hours)** | Environment | 0.110 (-1.058, 1.282) | -0.098 (-1.210, 1.047) | -0.131 (-1.416, 1.212) | 0.123 (-1.187, 1.485) | -0.205 (-1.426, 1.073) | 0.801 (-0.750, 2.413) |
|  | Physical | -1.242 (-2.581, 0.092) | **-1.524 (-2.704, -0.391)** | -1.533 (-2.826, 0.258) | **-1.377 (-2.702, -0.095)** | **-1.607 (-2.842, -0.392)** | -0.876 (-2.314, 0.535) |
|  | Psychological | -0.567 (-1.826, 0.689) | -0.621 (-1.833, 0.548) | -0.792 (-2.189, 0.579) | -0.313 (-1.682, 1.025) | -1.384 (-2.676, -0.088) | -0.595 (-2.138, 0.967) |
|  | Social | -0.181 (-2.059, 1.702) | -0.732 (-2.518, 1.076) | -0.868 (-2.88, 1.139) | -0.785 (-2.836, 1.291) | -1.135 (-3.082, 0.837) | -0.660 (-2.970, 1.685) |
| **Sleep efficiency**  **(%)** | Environment | 0.105 (-0.081, 0.291) | -0.067 (-0.243, 0.111) | -0.163 (-0.389, 0.067) | -0.086 (-0.317, 0.144) | -0.070 (-0.287, 0.146) | -0.042 (-0.299, 0.216) |
|  | Physical | 0.212 (-0.002, 0.429) | 0.013 (-0.167, 0.192) | 0.068 (-0.150, 0.287) | 0.0827 (-0.143, 0.303) | 0.0811 (-0.131, 0.292) | 0.071 (-0.158, 0.302) |
|  | Psychological | 0.083 (-0.117, 0.285) | -0.056 (-0.240, 0.131) | 0.063 (-0.178, 0.303) | 0.031 (-0.204, 0.260) | 0.029 (-0.195, 0.253) | 0.081 (-0.171, 0.332) |
|  | Social | 0.209 (-0.078, 0.497) | -0.013 (-0.289, 0.263) | -0.068 (-0.409, 0.280) | -0.004 (-0.355, 0.345) | -0.021 (-0.356, 0.314) | -0.009 (-0.385, 0.372) |
| **Fragmentation index** | Environment | -0.000 (-0.099, 0.098) | 0.062 (-0.033, 0.159) | 0.112 (-0.002, 0.226) | 0.086 (-0.027, 0.200) | 0.061 (-0.046, 0.171) | 0.065 (-0.065, 0.193) |
|  | Physical | -0.176 (-0.291, -0.062) | -0.055 (-0.150, 0.044) | -0.045 (-0.153, 0.067) | -0.075 (-0.184, 0.036) | -0.057 (-0.161, 0.050) | -0.054 (-0.168, 0.063) |
|  | Psychological | -0.012 (-0.119, 0.094) | 0.060 (-0.040, 0.161) | 0.039 (-0.079, 0.161) | 0.093 (-0.021, 0.207) | 0.060 (-0.051, 0.173) | 0.069 (-0.057, 0.193) |
|  | Social | -0.103 (-0.256, 0.049) | -0.002 (-0.152, 0.148) | 0.056 (-0.119, 0.227) | 0.008 (-0.165, 0.181) | -0.012 (-0.180, 0.156) | 0.020 (-0.170, 0.208) |
| WHOQoL-BREF - World Health Organization Quality of Life Brief  All results are β (95% confidence interval). Results which exclude zero are highlighted in bold  *The relationship between Consensus Sleep Diary average scores and HRQoL  1 Age, sex, Index of Multiple Deprivation, smoking, alcohol, marital status, number of medications, body mass index (self-reported kg/m2), Hospital Anxiety and Depression anxiety scale, RAPID-3, Obstructive Sleep Apnea, and Restless Leg Syndrome  2 As (1) above plus iSD and autocorrelation measures of sleep parameter, pain, mood and fatigue | | | | | | | |

| **Table S4. Sleep actigraph and WHOQoL-BREF domains** | | | | | | | | | | |
| --- | --- | --- | --- | --- | --- | --- | --- | --- | --- | --- |
| **Sleep parameter** | **Quality of life domain** | **Sleep parameter measure** | **Univariable** | **Multivariable** | | | **R^2^ univariable** | | **R^2^ multivariable**  **(all covariates)** | |
|  |  |  |  | Baseline factors^1^ | Sleep variability^2^ | All co-variates^3^ | Marginal | Conditional | Marginal | Conditional |
|  | | | | | | | | | | |
| **Sleep onset latency**  **(Hours)** | Environment | Average | -0.06 (-0.13,0.01) | 0 (-0.07,0.07) | 0.07 (-0.03,0.16) | 0.03 (-0.1,0.16) | 0 | 0.89 | 0.41 | 0.87 |
|  |  | iSD |  |  | -0.09 (-0.19,0) | -0.11 (-0.25,0.03) |  |  |  |  |
|  |  | Autocorr. |  |  | 1.81 (-0.17,3.8) | 2.07 (-0.73,4.92) |  |  |  |  |
|  | Physical | Average | -0.07 (-0.15,0.01) | -0.02 (-0.09,0.05) | -0.04 (-0.13,0.07) | -0.1 (-0.22,0.03) | 0 | 0.91 | 0.66 | 0.91 |
|  |  | iSD |  |  | 0.03 (-0.08,0.13) | 0.09 (-0.04,0.22) |  |  |  |  |
|  |  | Autocorr. |  |  | -2.09 (-4.26,0.07) | -2.22 (-4.94,0.54) |  |  |  |  |
|  | Psychological | Average | -0.05 (-0.12,0.03) | -0.01 (-0.09,0.06) | -0.09 (-0.19,0.01) | -0.2 (-0.33,-0.07) | 0 | 0.90 | 0.52 | 0.89 |
|  |  | iSD |  |  | 0.11 (0.01,0.21) | 0.23 (0.1,0.37) |  |  |  |  |
|  |  | Autocorr |  |  | -0.2 (-2.32,1.93) | -1.51 (-4.27,1.32) |  |  |  |  |
|  | Social | Average | -0.06 (-0.17,0.04) | 0.01 (-0.09,0.12) | 0.05 (-0.1,0.21) | 0 (-0.19,0.21) | 0 | 0.81 | 0.29 | 0.82 |
|  |  | iSD |  |  | -0.04 (-0.2,0.12) | 0.01 (-0.2,0.22) |  |  |  |  |
|  |  | Autocorr. |  |  | -2.67 (-6.08,0.72) | 0.51 (-3.95,4.9) |  |  |  |  |
|  | | | | | | | | | | |
| **Sleep efficiency**  **(%)** | Environment | Average | 0.1 (-0.08,0.29) | -0.07 (-0.24,0.11) | -0.15 (-0.35,0.06) | -0.04 (-0.3,0.22) | 0 | 0.89 | 0.40 | 0.88 |
|  |  | iSD |  |  | -0.21 (-0.5,0.09) | -0.28 (-0.69,0.12) |  |  |  |  |
|  |  | Autocorr. |  |  | -1.44 (-3.43,0.55) | -2.21 (-5.04,0.64) |  |  |  |  |
|  | Physical | Average | 0.21 (0,0.43) | 0.01 (-0.17,0.19) | 0.05 (-0.16,0.25) | 0.07 (-0.16,0.3) | 0 | 0.91 | 0.66 | 0.9 |
|  |  | iSD |  |  | 0.11 (-0.21,0.43) | 0.02 (-0.36,0.42) |  |  |  |  |
|  |  | Autocorr. |  |  | -1.31 (-3.47,0.87) | **-3.32 (-6.12,-0.59)** |  |  |  |  |
|  | Psychological | Average | 0.08 (-0.12,0.28) | -0.06 (-0.24,0.13) | 0.02 (-0.19,0.24) | 0.08 (-0.17,0.33) | 0 | 0.90 | 0.51 | 0.89 |
|  |  | iSD |  |  | 0.26 (-0.05,0.57) | 0.38 (-0.03,0.78) |  |  |  |  |
|  |  | Autocorr. |  |  | -1.11 (-3.22,1.03) | -1.38 (-4.2,1.51) |  |  |  |  |
|  | Social | Average | 0.21 (-0.08,0.5) | -0.01 (-0.29,0.26) | -0.02 (-0.34,0.3) | -0.01 (-0.39,0.37) | 0 | 0.81 | 0.29 | 0.82 |
|  |  | iSD |  |  | 0.01 (-0.47,0.51) | 0.06 (-0.56,0.69) |  |  |  |  |
|  |  | Autocorr. |  |  | 0.24 (-3.14,3.64) | 0.09 (-4.27,4.55) |  |  |  |  |
|  | | | | | | | | | | |
| **Total time asleep**  **(Hours)** | Environment | Average | 0.01 (-0.22,0.25) | -0.14 (-0.37,0.08) | -0.25 (-0.51,0.01) | -0.21 (-0.53,0.11) | 0 | 0.89 | 0.40 | 0.88 |
|  |  | iSD |  |  | -0.24 (-0.59,0.11) | -0.46 (-0.94,0.03) |  |  |  |  |
|  |  | Autocorr. |  |  | -1.46 (-3.46,0.5) | -1.31 (-4.18,1.49) |  |  |  |  |
|  | Physical | Average | 0.25 (-0.02,0.53) | 0.05 (-0.18,0.28) | 0.10 (-0.17,0.36) | 0.11 (-0.17,0.4) | 0 | 0.91 | 0.66 | 0.90 |
|  |  | iSD |  |  | 0.14 (-0.23,0.52) | 0.01 (-0.45,0.48) |  |  |  |  |
|  |  | Autocorr. |  |  | -1.13 (-3.26,1.06) | -2.55 (-5.3,0.22) |  |  |  |  |
|  | Psychological | Average | -0.01 (-0.27,0.24) | -0.15 (-0.38,0.08) | -0.1 (-0.37,0.18) | -0.01 (-0.33,0.3) | 0 | 0.9 | 0.51 | 0.89 |
|  |  | iSD |  |  | 0.18 (-0.19,0.55) | 0.31 (-0.18,0.79) |  |  |  |  |
|  |  | Autocorr. |  |  | -2.17 (-4.3,-0.07) | -2.47 (-5.32,0.37) |  |  |  |  |
|  | Social | Average | 0.19 (-0.18,0.56) | -0.02 (-0.36,0.33) | -0.10 (-0.5,0.32) | -0.05 (-0.52,0.43) | 0 | 0.81 | 0.29 | 0.82 |
|  |  | iSD |  |  | -0.19 (-0.76,0.41) | -0.14 (-0.86,0.63) |  |  |  |  |
|  |  | Autocorr. |  |  | -0.61 (-4.00,2.76) | -1.05 (-5.47,3.32) |  |  |  |  |
|  | | | | | | | | | | |
| **Fragmentation index** | Environment | Average | 0 (-0.1,0.1) | 0.06 (-0.03,0.16) | 0.08 (-0.02,0.19) | 0.06 (-0.06,0.19) | 0 | 0.89 | 0.4 | 0.87 |
|  |  | iSD |  |  | -0.09 (-0.25,0.06) | -0.1 (-0.32,0.11) |  |  |  |  |
|  |  | Autocorr. |  |  | -1.12 (-3.01,0.73) | -1.34 (-4.26,1.53) |  |  |  |  |
|  | Physical | Average | **-0.18 (-0.29,-0.06)** | -0.05 (-0.15,0.04) | -0.06 (-0.16,0.05) | -0.05 (-0.17,0.06) | 0.01 | 0.91 | 0.66 | 0.9 |
|  |  | iSD |  |  | 0.03 (-0.14,0.19) | 0.04 (-0.17,0.24) |  |  |  |  |
|  |  | Autocorr. |  |  | -1.83 (-3.86,0.21) | -1.56 (-4.34,1.3) |  |  |  |  |
|  | Psychological | Average | -0.01 (-0.12,0.09) | 0.06 (-0.04,0.16) | 0.05 (-0.05,0.16) | 0.07 (-0.06,0.19) | 0 | 0.9 | 0.51 | 0.89 |
|  |  | iSD |  |  | 0.04 (-0.12,0.21) | 0.1 (-0.12,0.31) |  |  |  |  |
|  |  | Autocorr. |  |  | -2.38 (-4.36,-0.37) | -1.33 (-4.17,1.63) |  |  |  |  |
|  | Social | Average | -0.1 (-0.26,0.05) | 0 (-0.15,0.15) | 0 (-0.16,0.16) | 0.02 (-0.17,0.21) | 0 | 0.81 | 0.29 | 0.82 |
|  |  | iSD |  |  | -0.02 (-0.28,0.24) | 0.03 (-0.29,0.37) |  |  |  |  |
|  |  | Autocorr. |  |  | -0.71 (-3.89,2.54) | 0.22 (-4.22,4.77) |  |  |  |  |
|  | | | | | | | | | | |
| WHOQoL-BREF - World Health Organization Quality of Life Brief  All results are β (95% confidence interval). Results which exclude zero are highlighted in bold.  iSD = intra-individual Standard Deviation. Higher values indicate higher amplitude of fluctuations.  Autocorrelation (temporal dependency) assesses the extent to which observations can be predicted from previous observations. Positive values up to 1 indicate stable predictor variable reports (above or below mean value) with little variation with higher values indicating increased stability. Values of 0 indicate low predictive ability. Negative values up to -1 indicate fluctuating predictor variable reports (i.e. if the value of the predictor variable is below the mean, the next value is likely to be above the mean; if the value of the predictor variable is above the mean the next value is likely to be below the mean) with lower values indicating increased fluctuations.  1 Age, sex, Index of Multiple Deprivation, smoking, alcohol, marital status, number of medications, body mass index (self-reported kg/m2), Hospital Anxiety and Depression anxiety scale, RAPID-3, Obstructive Sleep Apnea, and Restless Leg Syndrome  2 As (1) above plus sleep parameter iSD and Autocorrelation  3 As (2) above plus average, iSD and autocorrelation measures of pain, mood and fatigue | | | | | | | | | | |
